# IPA and WPA-SOAP Strategies to Promote the Human Rights in Mental Health Care of Older Adults

**DOI:** 10.1192/j.eurpsy.2022.126

**Published:** 2022-09-01

**Authors:** C.A. De Mendonça Lima

**Affiliations:** Centre Médical du Jorat, Psychiatry, Mézières, Switzerland

**Keywords:** Old Age Mental Health, Human Rights

## Abstract

The number of persons with 60 years and more worldwide is estimated to triple by 2050. With the raising burden of the mental health conditions that accompany population ageing, mental health care for older adults has to be under pined by a dignity and human rights based approach. The extraordinary number of human rights violations of the older population during the COVID-19 pandemic has come to the forefront, as consequence of this population vulnerability, the lack of political will to give prior attention to this group needs and the disseminated ageistic attitudes. Discrimination based on age can lead to catastrophic social consequences such as elder abuse, neglect and all forms of violences. Their access to services become reduced, including health, social and justice services. These negative attitutdes, more than only morally unacceptable, are source of unnecessary suffering and increase morbidity and mortality rate. Intersecting across psychiatric diagnoses and interventions are the principles of dignity, autonomy, respect and equality which are all at the base of the call for an United Nations Convention of Rights of the Older People. Keeping all thes points in mind, the World Pschiatric Association Section of Old Age Psychiatry and the International Psychogeriatric Association are working together to promote the Human Rights of Older Adults. The presentation of a webinar, the publication of joint position statements, the organization of symposia in several international congress and the publication of a recent special issue of the America Jornal of Geriatric Psychiatry (October 2021 - https://www.ajgponline.org/issue/S1064-7481(21)X0010-3) are some examples of this common effort.

**Disclosure:**

No significant relationships.

